# Where to deliver? Analysis of choice of delivery location from a national survey in India

**DOI:** 10.1186/1471-2458-8-29

**Published:** 2008-01-24

**Authors:** Amardeep Thind, Amir Mohani, Kaberi Banerjee, Fred Hagigi

**Affiliations:** 1 Department of Family Medicine, Department of Epidemiology and Biostatistics, University of Western Ontario, London, Canada; 2 Dept of Internal Medicine, Hospital of Saint Raphael, New Haven, USA; 3 Department of Obstetrics & Gynecology, Moolchand Hospital, New Delhi, India; 4 Department of Health Services, UCLA School of Public Health, Los Angeles, USA

## Abstract

**Background:**

In order to reduce maternal mortality, the Indian government has increased its commitment to institutional deliveries. We assess the determinants of home, private and public sector utilization for a delivery in a Western state.

**Methods:**

Cross sectional analyses of the National Family Health Survey – 2 dataset.

**Setting:**

Maharashtra state. The dataset had a sample size of 5391 ever-married females between the ages of 15 to 49 years. Data were abstracted for the most recent birth (n = 1510) and these were used in the analyses. Conceptual framework was the Andersen Behavioral Model. Multinomial logistic regression analyses was conducted to assess the association of predisposing, enabling and need factors on use of home, public or private sector for delivery.

**Results:**

A majority delivered at home (n = 559, 37%); with private and public facility deliveries accounting for 32% (n = 493) and 31% (n = 454) respectively. For the choice set of home delivery versus public facility, women with higher birth order and those living in rural areas had greater odds of delivering at home, while increasing maternal age, greater media exposure, and more then three antenatal visits were associated with greater odds of delivery in a public facility. Maternal and paternal education, scheduled caste/tribe status, and media exposure were statistically significant predictors of the choice of public versus private facility delivery.

**Conclusion:**

As India's economy continues to grow, the private sector will continue to expand. Given the high household expenditures on health, the government needs to facilitate insurance schemes or provide grants to prevent impoverishment. It also needs to strengthen the public sector so that it can return to its mission of being the safety net.

## Background

Despite the Registrar General reporting recently that the maternal mortality rate in India declined to 301 per 100,00 live births, India still accounts for the largest contribution to maternal deaths worldwide, related directly to or exacerbated by pregnancy. [1-3] Reducing the maternal mortality rate is a key goal of government, and this is enshrined in the National Population Policy, which aims to "provide.... universal access to, and make available good quality maternal and child health care services".[4] One strategy advocated by the National Population Policy to reduce maternal mortality is to increase access to institutional deliveries in India.

The choice of delivery locations in India can be broadly be classified into three mutually exclusive categories. A woman can deliver her baby at home, with or without the presence of a birth attendant, who may be trained or untrained. Home delivery is usually the cheapest option, but is associated with attendant risks of infection and lack of available equipment should complications occur. Institutional deliveries can occur at private or public facilities. Public facilities are usually owned and financed by the government, and while costs are usually minimal, available amenities often leave much to be desired. Although private facilities are the most expensive, they are often perceived as having the best amenities and offering the best standard of care in India.

Most research has focused on understanding the determinants of home vs. institutional deliveries. [5-8] With rapid economic growth, there has been a concomitant expansion of the private sector in health care delivery in India. It is thus important to tease out the two types of institutions – public and private – as research has shown that each sector may provide different sets of services. For example, public hospitals provide a majority of hospitalizations, while the private sector provides most of the out-patient care.[9] Till date, there is no research that delineates the differences (if any) among these sectors in the provision of delivery care.

The purpose of the present study is to understand the determinants of delivery location (home, public or private facility) in one state of India (Maharashtra), using data from the Second National Family Health Survey (NFHS-2).

## Methods

### Research questions

What are the determinants of delivery location (home, public or private facility) the state of Maharashtra?

### Study design

The study was a cross sectional analyses of the National Family Health Survey – 2 (NFHS – 2) dataset.

### Data source

The NFHS – 2 is a house-to-house interview survey conducted in 1998–99 across 26 states of India. It interviewed a nationally representative sample of approximately 91,000 women.[10] The survey used a comprehensive questionnaire to interview ever-married females between 15 and 49 years and obtained in-depth information about family planning, infant and child mortality, maternal and child health, and utilization of reproductive and child health services by mothers and children.

The Maharashtra sample of the NFHS-2 data had a sample size of 5391 ever-married females between the ages of 15 to 49. We abstracted data from the most recent birth (n = 1510).

### Conceptual model

Andersen's Behavioral Model of Health Services Utilization was used as the conceptual framework.[11] This model has been used extensively in both developing and developed countries to understand health services utilization. [12-18] The model classifies factors that affect health services utilization into three groups: predisposing, enabling and need factors. Among the *predisposing *factors, demographic characteristics (age, gender, marital status) reflect the propensity of individuals to use services. Social structure (education, occupation, race/ethnicity) measures the ability of the individual to cope with the problem, the resources available in the community, and the state of the physical environment. Health beliefs are values and knowledge about health and the health care system that influence utilization, and these include general attitudes towards medical care, physicians, and disease.[11]

*Enabling *factors, both personal and organizational, must be present for service utilization, and these represent the actual ability of the individual to obtain health services. Personal enabling factors include income, health insurance, regular source of care, and travel and waiting times; organizational enabling factors include the availability of health care providers and their spatial distribution.[11] The most immediate cause of health services utilization is *need*. This judgment about need can be made by the individual himself or family caregivers (perceived need), and can be estimated by a self assessment of health status, symptoms experienced during a period of time, or number of symptoms during a period of time. Need can also be defined through a professional evaluation (evaluated need); for example, physician severity ratings for an episode of illness.[11]

### Variable specification

#### Dependent variable

A categorical dependent variable was created based on the location of the most recent live birth. It was categorized as 'home' if the mother reported that the most recent live birth occurred at home; 'public' if the most recent live birth occurred in a government hospital, government dispensary, urban health center, urban family welfare clinic, community health center or primary health center, and 'private' if the birth occurred at a private hospital, private clinic, and non-governmental organizations (NGO) or trust hospital. NGO and trust hospitals were included in the private sector category and could not be classified as a separate entity due to their small numbers.

#### Independent variables

The predisposing variables included in the model were birth order of the baby for which care was sought, sociodemographics of the mother and father (maternal age, maternal and paternal education, religion, and scheduled caste/tribe status). Given the emphasis placed till the recent past on the two child per family policy, we dichotomized birth order (two or less and more than two). Maternal age was a three level categorical variable (less than 20 years, 20 – 29 years, and more than 30 years). Based on the highest level of education completed, the mother's and father's education were categorized into three levels (primary or less, up to secondary, higher than secondary). Religion was coded into three categories, viz. Hindu, Muslim and others. To assess the effect of caste on the health services utilization, the NFHS – 2 variable classifying the population into scheduled caste/tribe (SC/ST) and non-SC/ST was used.

The enabling variables were household standard of living, media exposure, health care decision-maker, number of antenatal visits and location. The household standard of living is provided in the NFHS – 2 dataset, and is calculated based on the family assets and possessions. It is indicative of the economic status of the family, and is coded as low, medium and high.[10] Media exposure was a continuous variable, and was the scored summing the weekly exposure of the mother to newspapers, radio and television. The health care decision maker was categorized as the mother, father, mother and father jointly, or someone else in the family.[19] We dichotomized the number of antenatal visits into three or less and more than three, and a binary (rural, urban) location variable was used to capture community characteristics.

Need was based on the past history of stillbirths or abortions. If the woman reported having had a stillbirth, induced or spontaneous abortion in the past, she was categorized has having a 'high' need, if she did not have any of these, she was categorized as having a 'low' need.

### Data analysis

Data analysis was carried out using Stata SE, Version 10.[20] The unit of analysis was an ever-married female, who had had a live-birth in the past three years in the state of Maharashtra, and who delivered her baby at home, public or private facility. Univariate and bivariate analysis for each independent variable was performed against the dependent variable to elicit the impact of each factor on the pattern of health services utilization in the population, without adjusting for the effect of other variables. The dependent variable being categorical, a multinomial logistic regression model was used to assess the effect of each variable independently on the dependant variable while controlling for the confounders. Multicollinearity and interaction effects were evaluated for the model. To adjust for clustering, the Huber-White sandwich estimator of variance was used. The Hausman and Small Hsiao tests were conducted to ensure that the 'independence of irrelevant alternatives (IIA)' assumption was met in our data.

## Results

### Descriptive analyses

Of the 1,510 women who sought delivery care among the 5,391 women interviewed in Maharashtra in the NFHS-2, a majority delivered at home (n = 559, 37%); with private and public facility deliveries accounting for 32% (n = 493) and 31% (n = 454) respectively. Table 1 illustrates the characteristics of the respondents. Among predisposing variables, a higher birth order, Hindu religion and scheduled caste/tribe status were associated with home delivery, while increasing maternal age, greater mother's and father's education, and a lower birth order was associated with use of private facilities.

**Table 1:** Descriptive characteristics of deliveries.

	Place of delivery		
	Home (n = 559)	Public facility (n = 454)	Private facility (n = 493)
*Predisposing*			
Birth order*			
2 or less	276 (30%)	292 (31.8%)	351 (38.2%)
More than 2	283 (48.2%)	162 (27.6%)	142 (24.2%)
Mother's age*			
< 20 yrs	365 (43.6%)	246 (29.4%)	226 (27%)
20 – 29 yrs	144 (31.7%)	140 (30.8%)	171 (37.5%)
> 30 yrs	50 (23.4%)	68 (31.8%)	96 (44.8%)
Mother's education*			
Primary or less	399 (55.9%)	206 (28.8%)	109 (15.3%)
Up to secondary	143 (25%)	199 (34.7%)	231 (40.3%)
More than secondary	17 (7.8%)	49 (22.4%)	153 (69.8%)
Father's education*			
Primary or less	270 (58%)	130 (28%)	66 (14%)
Up to secondary	218 (32%)	246 (36.2%)	216 (31.8%)
More than secondary	68 (19.3%)	76 (21.6%)	208 (59.1%)
Religion*			
Hindu	465 (42.7%)	300 (27.5%)	324 (29.6%)
Muslim	57 (20.4%)	103 (36.8%)	120 (42.8%)
Others	33 (25.6%)	49 (38%)	47 (36.4%)
Scheduled caste status*			
No	295 (32.7%)	256 (28.4%)	350 (38.9%)
Yes	260 (43.4%)	196 (32.7%)	143 (23.9%)
*Enabling*			
Household standard of living*			
Low	274 (71.3%)	73 (19%)	37 (9.7%)
Medium	238 (32.1%)	272 (36.7%)	231 (31.2%)
High	30 (9.8%)	83 (27%)	194 (63.2%)
Media exposure*	0.75	1.6^a^	2.0^a^
Healthcare decision-maker*			
Mother	133 (27.1%)	167 (34%)	191 (38.9%)
Father	280 (45.3%)	165 (26.7%)	174 (28.1%)
Mother & father jointly	54 (30.3%)	63 (35.4%)	61 (34.3%)
Others in household	92 (42.2%)	59 (27.1%)	67 (30.7%)
Number of antenatal visits*			
3 or less	451 (60.5%)	155 (20.8%)	139 (18.7%)
More than 3	107 (14.1%)	298 (39.3%)	354 (46.6%)
Location*			
Urban	130 (15.3%)	341 (40.1%)	380 (44.6%)
Rural	429 (65.5%)	113 (17.3%)	113 (17.2%)
*Need*			
Low	487 (36.8%)	401 (30.3%)	435 (32.9%)
High	72 (39.3%)	53 (29%)	58 (31.7%)

^a ^indicates a continuous variable; * p < 0.05

Among the enabling variables, a higher standard of living, greater media exposure, and greater number of antenatal visits were associated with use of private facilities for delivery. When the mother was the healthcare decision maker, the delivery was more likely to occur in a private facility. Respondents living in rural areas were more likely to deliver at home. Need was not statistically significantly associated with place of delivery.

### Multivariate analyses

Table 2 presents the results of multinomial logistic regression model. For the choice set of home delivery versus public facility, women with higher birth order and those living in rural areas had greater odds of delivering at home compared to using public facilities; while increasing maternal age, greater media exposure, and more then three antenatal visits were associated with greater odds of delivery in a public facility. Compared to Hindu women, Muslim women had lesser odds of delivering at home. The same predictors were statistically significant determinants of the choice set of home delivery versus private facility; additionally, the household standard of living was also significant, with decreased odds of home deliveries in households with a high standard of living, compared to households with a low standard of living.

**Table 2:** Multinomial logistic regression analysis of the odds of delivery at home, public or private facility (n = 1410).

	Odds Ratio		
	Home vs. Public	Home vs. private	Public vs. private
*Predisposing*			
Birth order			
2 or less	-	-	-
More than 2	1.58*	1.81*	1.14
Mother's age			
< 20 yrs	-	-	-
20 – 29 yrs	0.64*	0.50*	0.77
> 30 yrs	0.60*	0.40*	0.67
Mother's education			
Primary or less	-	-	-
Up to secondary	0.82	0.48*	0.59*
More than secondary	1.11	0.37*	0.34*
Father's education			
Primary or less	-	-	-
Up to secondary	0.72	0.58*	0.80
More than secondary	1.01	0.40*	0.40*
Religion			
Hindu	-	-	-
Muslim	0.57*	0.50*	0.88
Others	0.83	1.12	1.35
Scheduled caste status			
No	-	-	-
Yes	0.76	1.36	1.80*
*Enabling*			
Household standard of living			
Low	-	-	-
Medium	0.94	0.82	0.87
High	0.77	0.42*	0.55*
Media exposure	0.62*	0.60*	0.97
Healthcare decision-maker			
Mother	-	-	-
Father	1.34	1.04	0.78
Mother & father jointly	0.88	1.03	1.16
Others in household	1.19	1.13	0.95
Number of antenatal visits			
3 or less	-	-	-
More than 3	0.26*	0.30*	1.18
Location			
Urban	-	-	-
Rural	4.82*	4.85*	1.01
*Need*			
Low	-	-	-
High	1.03	0.99	0.96

* p < 0.05

Maternal and paternal education, scheduled caste/tribe status, and media exposure were statistically significant predictors of the choice of public versus private facility delivery. Compared to women with primary education or less, women who had up to secondary level education had 41% lower odds of using a public facility, while women who had more than secondary education had 66% lesser odds of using a public facility. A similar trend was noted for paternal education – fathers with than secondary education had 60% lesser odds of using a public facility. Respondents belonging to scheduled caste/tribes were 80% more likely to deliver in public facilities; and women belonging to households with a high standard of living had 45% lower odds of using public facilities compared to women belonging to households with low standard of living.

## Discussion

The National Population Policy provides "a policy framework for advancing goals and prioritizing strategies during the next decade, to meet the reproductive and child health needs of the population of India".[4] The strategy involves focusing on the issues of child survival, maternal health, and contraception by increasing outreach, and addressing the unmet needs for care, infrastructure, and health personnel. Of the set of 14 socio – demographic goals for 2010, the chief reproductive and child health (RCH) related targets are reduction of the Infant Mortality Rate to below 30 per 1000 live births, and reduction of Maternal Mortality Ratio to below 100 per 100,000 live births. One strategy outlined in the NPP to reduce maternal mortality is to promote institutional delivery by strengthening the public system from the peripheral sub-center level to the referral unit level. Another strategy is to increase the numbers and diversify the categories of health care providers, promoting the involvement of, and developing partnerships with, the private health care industry and voluntary non-government sector.[4]

India spends approximately 5% of its GDP on health care.[21] Despite the rapid growth of its economy in the recent years, public funding for the health care sector has not kept pace. According to a recent report of the National Commission on Macroeconomics and Health, households accounted for nearly 75% of all health care expenditures, with the government accounting for only 22%.[22] Even in the state of Maharashtra, considered to be one of the more socially and economically progressive states in India, the state health budget has not increased commensurate with the increase in population and demand. Maharashtra's annual rate of growth of total expenditures on health grew from 3.8% in 1993 – 98 to 6.5% in 1999 – 2003, yet the per capita health expenditure averaged only Rupees 1576 in 2001 – 02 (1 Rupee = approx. 0.022 USD), and only 24% of pregnant women had received adequate antenatal care (defined as 3 antenatal care visits).[21]

There is increasing concern regarding the unmet need for care, especially among the destitute and the rural population. Consequently, the dependence on private sector for health care is increasing, more by default rather than by design. This is reflected in the fact that households in Maharashtra account for 73.3% of all health care spending, and the government accounted for 22.1% – percentages that mimic the all-India averages.[22] The dependence on household expenditures is equally significant in the reproductive and child health care segment, which is one of the high priority sectors of health care services in India.

The 63% institutional delivery rate in Maharashtra is much higher than the all India average for institutional deliveries, which is around a third.[8,10] Of the institutional deliveries in Maharashtra, almost equal numbers took place in public and private facilities, same as the national percentages. The discrepancy in the number of institutional deliveries can partly be explained by the fact that Maharashtra is one of the more economically developed states in India, and has relatively higher levels education, industrialization, and private sector growth. Moreover, the private sector share of the health care market in Maharashtra has been high. Its publicly funded rural health care delivery infrastructure is much better than that of many other states.

Our analyses show that among the predisposing factors, birth order, maternal education and religion affect the choice between home and public/private deliveries. It is plausible that after the uneventful birth of the first child at home, subsequent deliveries are perceived to be of low risk, thus increasing the likelihood of delivering subsequent babies at home. Increasing maternal age may, on the other hand, may increase the perception of risk, thus increasing the likelihood of institutional (public or private) delivery. Further research is need to understand why Muslims are less likely than Hindus' to deliver at home in Maharashtra.

Maternal and paternal education, and scheduled caste status are the predisposing factors that determine the choice between private facilities and public/home deliveries. The direction of the education effect is in the expected direction – increasing education leading to increased probability of using private facilities. Education leads to better health awareness, and this may sensitize the family to the quality of health care provided at various facilities.[5,23] It is a common perception in many parts of the country that private facilities provide better care than public facilities.[6,8] Unfortunately, the lack of quality indicators in the present data set precludes further analyses of this issue. Women from scheduled caste/scheduled tribes were less likely to use private sector facilities, which may be am indication of the continued exclusion of these groups from the mainstream. Despite vigorous efforts to integrate these dispossessed into society, much work needs to be done especially in the rural parts of the country.

Among the enabling factors, media exposure, number of antenatal visits and location affected the choice between home and private/public facilities, while a higher standard of living affected the choice between private and home/public facilities. The rich have the wherewithal to pay for the price of private care, and given the perception of better quality at private facilities, it is no surprise that they utilize them.

We did not find need to be a significant determinant of public versus private sector use. The Andersen Model posits that need is the most proximate determinant of health services utilization. Need can be conceptualized as perceived (by the individual woman) or evaluated (by an expert, such as a physician); our construction of need is more evaluative than perceived. It is possible that perceived need may be the appropriate variable to use, however, the NFHS-2 lacks sufficient data to construct such a variable.

Policy recommendations from our analyses are threefold. First, given the preponderance of home deliveries in the state, especially in the rural areas, every attempt should be made to ensure that these are attended to by a trained birth attendant. Efforts should also be made to ensure that every pregnant women receives at least 3 antenatal visits, with each visit being used as an educational opportunity. This requires strengthening the public health and outreach components of the publicly funded health care system.

Second, private sector health care delivery system is a fact of life in Maharashtra. As the India's economy continues to grow, its effects will filter down to all levels of society, thus raising the household income. As this occurs, the likelihood of increased utilization of the private sector will increase, leading to a further expansion of this sector. If we take the importance of education and standard of living as determinants of private sector use, we need to set up mechanisms that will prevent impoverishment of families as they seek care from this sector. Given the percentage of household expenditures devoted to health care, it is important that insurance schemes be set up to financially protect the consumers. This is already happening in the country, and needs to be strengthened more.

Third, quality of care in the public sector should be improved, so that it becomes an attractive place to obtain care. This is especially important for households of low and middle income, who maybe using the private sector due to a perceived lack of quality, and may reach financial distress quicker than households with higher standards to living. In other words, the public sector needs to return to its mission of being the safety net for the vulnerable. The government has recognized the limitations of its Family Welfare program in providing services, and has implemented a new Reproductive and Child health program to deliver and even wider range of services.[24] The National Rural Health Mission and the 'Janani Suraksha Yojana' which aims to strengthen the community health centers are steps in the right direction, especially as they aim to devolve decision making to the panchayat level, which is closest to the users. [25-27]

A few caveats should be kept in mind regarding our study. It is limited to Maharashtra, and may not be generalizable to other states in the country, especially poorer states such as Bihar, where the context and environment may be quite different. Our analytic model does not take into account factors such as distance and travel time, which may affect the decision to use a particular facility. We do not explicitly control for the perceived quality of care in our model. Lastly, we categorize all private and all public providers into one category each, when in fact each group encompasses a heterogeneous range of providers, each of which may have distinct clientele.

## Conclusion

In conclusion, our study deepens our understanding of delivery location in one state of India, but further research is needed to be able to generalize the results to the rest of the country.

## Competing interests

The author(s) declare that they have no competing interests.

## Authors' contributions

AT was responsible for conceptualizing the research question, data acquisition and analyses, and drafting the manuscript. AM assisted in data analysis and manuscript drafting. KB assisted in data analyses and provided contextual and policy input. FH contributed to study design and help in manuscript drafting. All authors read and approved the final document.

## Pre-publication history

The pre-publication history for this paper can be accessed here:


